# Renal Transplants from Older Deceased Donors: Use of Preimplantation Biopsy and Differential Allocation to Dual or Single Kidney Transplant according to Histological Score Has No Advantages over Allocation to Single Kidney Transplant by Simple Clinical Indication

**DOI:** 10.1155/2018/4141756

**Published:** 2018-05-16

**Authors:** Costanza Casati, Valeriana Giuseppina Colombo, Marialuisa Perrino, Ornella Marina Rossetti, Marialuisa Querques, Alessandro Giacomoni, Agnese Binaggia, Giacomo Colussi

**Affiliations:** ^1^Division of Nephrology, Dialysis and Kidney Transplantation, ASST Grande Ospedale Metropolitano Niguarda, Milan, Italy; ^2^Division of Transplant Surgery, ASST Grande Ospedale Metropolitano Niguarda, Milan, Italy

## Abstract

**Background:**

Grafts from elderly donors (ECD) are increasingly allocated to single (SKT) or dual (DKT) kidney transplantation according to biopsy score. Indications and benefits of either procedure lack universal agreement.

**Methods:**

A total of 302 ECD-transplants in period from Jan 1, 2000, to Dec 31, 2015, were allocated to SKT (SKT_pre_) on clinical grounds alone (before Dec 2010, pre-DKT era, *n* = 170) or according to a clinical-histological protocol (after Dec 2010, DKT era, *n* = 132) to DKT (*n* = 48), SKT biopsy-based protocol (“high-risk”, SKT_hr_, *n* = 51), or SKT clinically based protocol (“low-risk”, SKT_lr_, *n* = 33). Graft and patient survival were compared between the two periods and between different transplant categories.

**Results:**

Graft and overall survival in recipients from ECD in pre-DKT and DKT era did not differ (5-year graft survival 87.7% and 84.2%, resp.); equal survival in the 2 ECD periods was shown in both donor age ranges of 60–69 and >70-years, and in low-risk or high-risk ECD categories. Within the DKT protocol SKT_hr_ showed worst graft and overall survival in the 60–69 donor age range; DKT did not result in significantly better outcome than SKT from ECD in either era. One-year posttransplant creatinine clearance in recipients did not differ between any ECD transplant category. At 3 and 5 years after transplantation there were significantly higher total dialysis-free recipient life years from an equal donor number in the pre-DKT era than in the DKT protocol.

**Conclusions:**

Use of a biopsy-based protocol to allocate grafts from aged donors to SKT or DKT did not result in better short term graft survival than a clinically based protocol with allocation only to SKT and reduced overall recipient dialysis-free life years in time.

## 1. Introduction

Organ shortage is nowadays the main constraint to kidney transplantation. In order to increase the donor pool and the chance of transplantation to patients on wait list, most transplant programs are increasingly accepting suboptimal, so called “extended criteria,” donors (ECD) [1, 2]. Transplants from ECD are known to perform worse than transplants from standard donors in terms of delayed graft function (DGF), primary nonfunction (PNF), short- and long-term renal function, and overall graft survival [1–3], yet they may offer a survival advantage in comparison with not being transplanted and remaining on wait list, at least for specific patient categories [4–6]. Thus, the decision whether to accept or discard ECD organs may be challenging. In some countries (including Italy), dual (DKT) rather than single (SKT) kidney transplantation from ECD has gained popularity as a means of limiting organ discard [7–10]; however, indications for differential allocation of ECD organs (two SKT versus DKT versus discard) are still ill defined and allocation criteria differ between centres [11–14]. It has been feared that DKT may reduce rather than increase the number of transplants from available donors and that use of organ biopsy to guide allocation decision may itself increase graft discard rate [15].

In our Renal Transplant Area (Nord Italia Transplant program, NITp), comprising 6 Northern Italy regions with a total of about 19 million inhabitants, a protocol for allocation of grafts from elderly donors to DKT or SKT according to biopsy indication has been available as an option to expand donor pool since early 2000s [8]. Our Centre joined the NITp DKT program in 2010; before that date, only SKT was considered for any donor, irrespective of age, provided that renal function and anatomy were permissive. The aim of present study was to prospectively evaluate results of transplant activity from ECD performed in our centre in the first 5 years after adoption of the DKT program (“DKT era”) in comparison with that performed before that date. Main focus was on graft survival; calculation of dialysis-free life years at different times after transplantation in recipients within the two protocols was also done.

## 2. Methods

### 2.1. Donor Categories and Transplant Types

The DKT program in our Centre complies with a protocol of the NITp, our interregional transplant agency [8], where it is publicly registered [16, 17]; the following definitions are in use.

“high-risk” ECD (ECD_hr_): it means donors older than 70 years, or aged 60–69 years with any of the following: arterial hypertension treated with ≥2 drugs, drug-treated diabetes mellitus, known coronary, cerebral, or peripheral vascular disease or death due to cerebrovascular event (with exclusion of hemorrhage from ruptured arterial aneurism and cardiac embolism), proteinuria higher than 0.5 g/l, and eGFR (Cockcroft-Gault) less than 60 ml/min/1.73 mq.

“low-risk ECD” (ECD_lr_): it means donors aged 60–69 years without any of the above comorbidities.

Organs from ECD_lr_ are allocated to SKT without biopsy (SKT_lr_); those from ECD_hr_ are allocated to SKT (SKT_hr_) if score is 0 to 4 and to DKT if score is 5 to 7 or discarded if score is higher than 7 according to biopsy (Table 1). DKT program is additive to the standard donor SKT program; only patients consenting to the program by written informed consent, and who are in our Centre and older than 62 years, are offered ECD_hr_ organs, for either DKT or SKT_hr_.

DKT program in our Centre started on Dec. 2010; from 1 Dec., 2010, to 31 Dec., 2015, 132 ECD (33 ECD_lr_ and 99 ECD_hr_) were allocated according to DKT protocol: 33 were SKT_lr_, 48 were DKT, and 51 were SKT_hr_ (Table 2).

Before adopting DKT program (“pre-DKT era”), organs from any donors were allocated only to SKT, provided that lower predonation plasma creatinine was normal and eGFR (Cockroft-Gault formula) was higher than 60 ml/min/1.73 m^2^, proteinuria was absent or “trace”, and anatomy was (echography and/or surgical) permissive. Comorbidities and cause of death were not specifically addressed to, if function was preserved. Preimplantation biopsy was considered only for cause, that is, to ascertain specific pathologies deemed by clinical context. A hundred seventy consecutive transplants (SKT_pre_) from ECD_pre_ were performed from 1 Jan, 2000, to 30 Nov, 2010, and were compared to transplants within the DKT protocol. A hundred and three of pre-DKT donors comply to “high-risk” definition according to the DKT protocol criteria and 48 to “low-risk” definition; for remaining 19 donors we had insufficient information for unequivocal categorization into either low or high-risk and they were not included in the intercategory comparisons.

All recipients were over 18 years of age. Database update ended on 30 Jun, 2017. Since only 11 patients in the DKT protocol had a longer than 6-year follow-up, all event-free patients in either protocol were right-censored at 6 years from transplant.

All donors were brain-dead; transplants from living, cardiac-death, or ABO- or HLA-incompatible donors, as well as combined solid organ transplants, were not included. Both first and nonfirst transplants were included. A pretransplant negative T and B-lymphocyte CDC was a prerequisite for transplantation; best HLA match was sought for allocation in all transplant categories except in DKT, with careful avoidance of any HLA forbidden antigen according to actual or historical circulating antibodies.

Informed consent was obtained from all the patients applying for renal transplantation in our Centre at the time of listing and at the time of transplantation, and additionally for applying to the DKT program. This study has been conducted according to principles of the declaration of Helsinki and complies with the declaration of Istanbul. As a standard of care-based, anonymous study, no approval by ethics committee was needed in our institution.

### 2.2. Study Outcomes

For every donor-recipient pair, in each donor category, we collected and analyzed age, sex, HLA mismatches (loci A, B, and DRB1), type and length of dialysis, plasma creatinine and eGFR of donor, plasma creatinine and creatinine clearance (24-hour urine) at 3 months and 1 year after transplant, and biopsy-proven rejection of any type in the first 18 months after transplantation in recipients. Main outcomes were death-censored graft survival (i.e., freedom from dialysis or retransplantation) and overall graft survival (including death as a cause of graft loss, i.e., patients alive with functioning graft); as secondary outcomes we also evaluated PNF (no dialysis-freedom, or need for permanent dialysis within 3 months after transplant), DGF (need for dialysis for any cause in the first week after transplant), patient death with functioning graft, and renal function in recipients at 3 and 12 months after transplant. Additionally, mean number of functioning graft years by transplant reference, and of dialysis-free life years by donor reference, were also calculated at specific times (see below).

### 2.3. Immunosuppression Protocols

Immunosuppression protocols at our Centre did not change in all observation period (Jan 2000 to Dec 2015) and included in most patients rATG induction (3.5 mg/Kg in 7 days, 7 mg/Kg if ≥2nd transplant), cyclosporine-A starting from pretransplantation as a 10 mg/Kg oral load, Mycophenolate mofetil/Mycophenolic acid starting on p.o. day 1 (1 g or 720 mg bid), and corticosteroids (methylprednisolone 500 mg at reperfusion, rapidly tapered down to 8 mg/day on p.o. day 11 and 4 mg/day after 3 months); a minority of patients (less than 10%), enrolled in clinical studies, might have been induced with Basiliximab and/or treated with Tacrolimus, Everolimus, or Sirolimus as alternatives; Azathioprine was also substituted for Mycophenolate in gastrointestinal intolerant patients. Posttransplant heparin anticoagulation was started in 2011 only in DKT, and after that a higher than usual graft vein thrombosis was observed in this type of transplant, as described also by others [18].

### 2.4. Statistics

Descriptive statistics are given as numbers, percentages, and mean (±SD) or median (and IQR) according to data distribution; intercategory differences were checked by ANOVA followed by Scheffé post hoc test; the chi-square method was used for comparison of frequencies of categorical data. Survival analysis was estimated as event-free cumulative survival using the Kaplan-Meier method and compared using the log-rank Mantel-Cox test. Cox regression analysis was used to calculate hazard ratios of cumulative incidence of events within each transplant category, and to calculate relative risks associated with patient and donor characteristics.

We estimated the mean number of years in which the allografts were functioning before loss for any cause (failure or death with functioning graft) by the restricted mean survival analysis [19–21]; it is computed as the total area under the survival curve at specific times (we repeated the procedure at 1, 3, and 5 posttransplant years). Conceptually, this procedure indicates the mean time (years) each graft remained functional at any defined time and equals the mean dialysis-free life years for every recipient at any defined time. From this value we extrapolated total dialysis-free life years for every 100 donors at any time in our pre-DKT and the DKT protocols; for this calculation each donor was made equal to 1.6 SKT (according to data of our regional agency on utilization of overall retrieved grafts) [22] or 1 DKT according to allocation.

SPSS Statistics software v.21 was used for all analyses. Two-tailed *P* values < 0.05 were considered significant.

## 3. Results

Summary data of all ECD and transplant categories are given in Table 2: donor sex distribution did not differ within each transplant category; donor age was similar in “low-risk” donors of either era, while it was statistically different between each of “high-risk” transplant categories. A hundred and seven donors were older than 70 years: 75 in the DKT era, allowing 43 DKT and 32 SKT_hr_, and 32 in pre-DKT period; mean age in donors aged more than 70 years was slightly lower in pre-DKT donors than in donors of DKT category. There were no major differences in comorbidity type and incidence between all high-risk donor categories, except lower incidence of cerebrovascular cause of death in SKT_hr_.

Recipient characteristics in each transplant category and main transplant outcomes are shown in Table 3: age increased stepwise according to donor category, reflecting the general rule of D-R matching by age. DKT recipients had higher HLA mismatches than all other transplant categories, reflecting allocation policy to DKT program. Mean and total follow-up was lower in all categories of the DKT era by selection. Graft cold ischemia time to transplant was longer in SKT_hr_ and DKT than in SKT_pre_ and SKT_lr_, possibly due to biopsy processing times.

PNF from any cause occurred in 8 of 170 SKT_pre_ and in 9 of 132 transplants in the DKT program (*P* = NS, Table 3). Use of biopsy apparently did not decrease PNF risk in SKT from ECD: in donors aged more than 70 years, there were 4 cases of 32 (12.5%) in pre-DKT era without biopsy (SKT_pre_) and 4 of 32 (12.5%) in the DKT protocol with biopsy (SKT_hr_); in donors aged 60–69 years, there were 5 cases of 171 transplants (138 SKT_pre_ and 33 SKT_lr_) without biopsy (3.0%) and 2 cases of 19 with biopsy (SKT_hr_) (10,5%; *P* < 0.05).

Preimplantation biopsy score itself was not predictive of PNF in SKT_hr_: score 4 (the highest for SKT allocation) was present in 4 of 6 SKT_hr_ with PNF and in 31 of 44 without PNF (*P* = NS); PNF occurred in 4 of 35 score 4 grafts (11.4%), 1 of 8 score 3 (12.5%), and 1 of 8 score 1 or 2 (12.5%) (*P* = NS).

Posttransplant histology in 9 nonsurgical PNF showed acute rejection (humoral and cellular) in only 1 SKT_pre_; in all other cases it showed interstitial fibrosis, severe tubular damage and atrophy, and arteriosclerosis as main features, indicating poor organ quality; additional findings were patchy necrotic areas (one with atheroembolism) in 2 cases (1 SKT_pre_ and 1 SKT_hr_), suggesting embolism from manipulated recipient vessels, and diabetic glomerulosclerosis (1 SKT_hr_) which had been missed in the preimplantation biopsy.

Rejection of any type (cellular and/or humoral) in biopsies performed for cause in the first 18 months after transplant was statistically higher in SKT_hr_ than in SKT_pre_ (*P* < 0.05) (Table 3); however rejection was not associated with graft and overall survival in multivariate Cox analysis (data not shown).

Death with functioning graft was not statistically different in any transplant category; main causes of death were neoplasia (27% pre-DKT and 29% post-DKT, resp.), infection (20 and 21%), cardiovascular diseases (29 and 36%), and other (24 and 14%) (all *P* = NS).

### 3.1. Survival Analysis by Period and Donor Category

**Figure 1:**
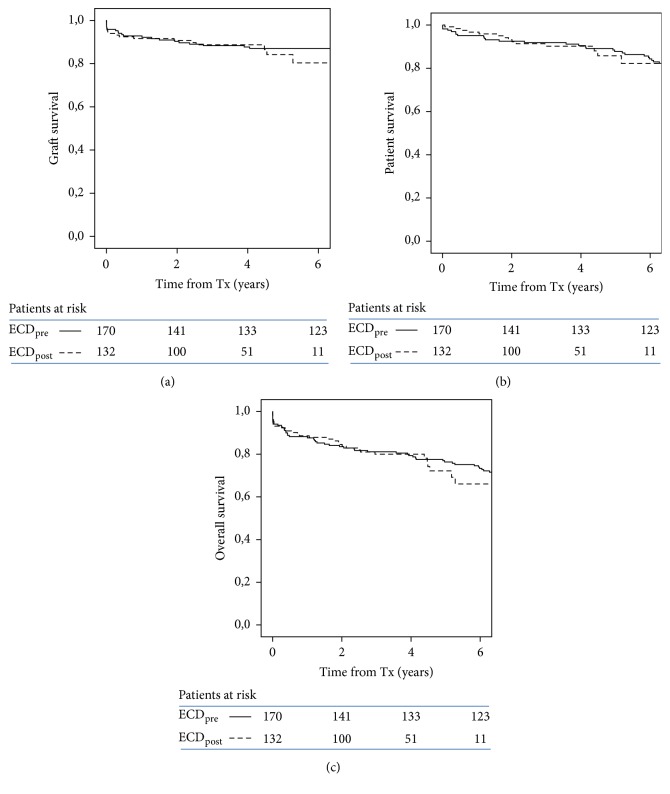
Kaplan-Meier plots of graft (death-censored) (a), patient (b), and overall survival (c) in transplant recipients of ECD organs according to period (ECD_pre_: pre-DKT period; ECD_post_: DKT era).

There were no statistically significant differences in graft, patient, and overall survival between transplants from either the pre-DKT or the DKT periods (Figure 1). Five-year graft survival was 87.7% in SKT_pre_ and 84.2% in all the DKT era transplants.

**Figure 2:**
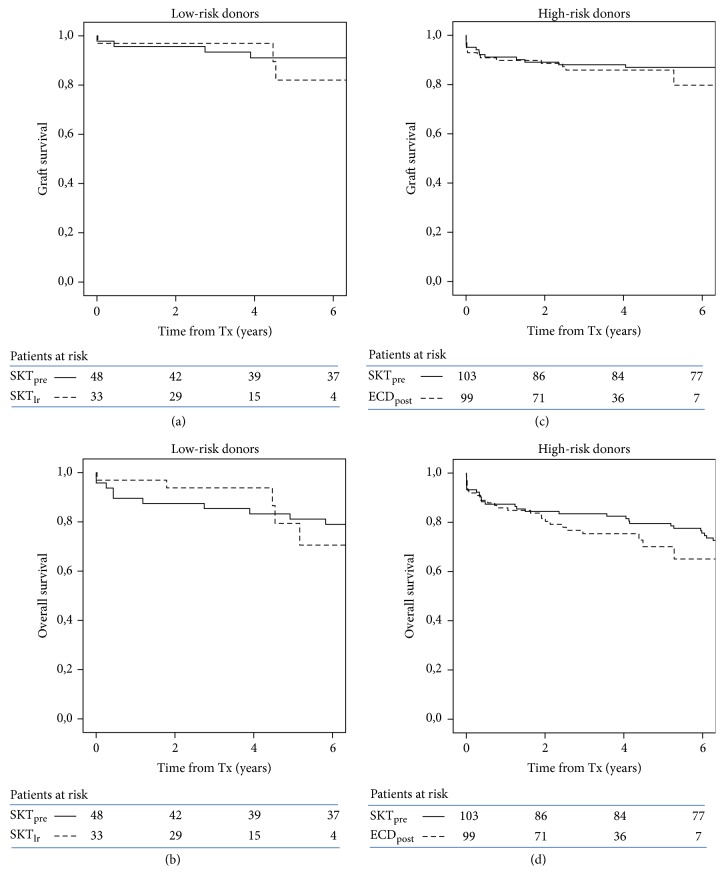
Kaplan-Meier plots of graft (a, c) and overall survival (b, d) in transplants by donor category (low-risk and high-risk) and period (SKT_pre_: pre-DKT donors; SKT_lr_ and ECD_post_: DKT protocol). ECD_post_ includes SKT_hr_ and DKT. All comparisons are statistically not significant.

Survival analysis by donor risk category (low-risk and high-risk) showed again equal graft and overall survival between pre-DKT period and DKT era (Figure 2).

**Figure 3:**
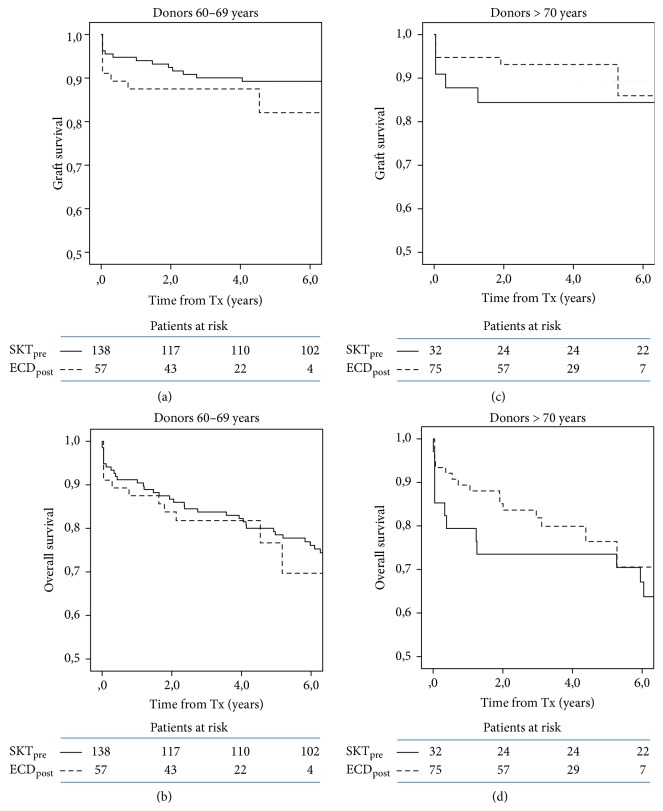
Kaplan-Meier plots of graft (a, c) and overall survival (b, d) in transplants from ECD according to donor age and period. ECD_post_ includes SKT_lr_ (60–69 age range), SKT_hr_, and DKT (donors > 70 years). All comparisons are statistically not significant.

To account for older donor age in the DKT era, transplants from donors aged 60 to 69 years and older than 70 years were separately evaluated: again, equal graft and overall survival were observed in recipients of the two eras from both donor age categories (Figure 3). In this analysis donor age was equal in low-risk donors of the two periods (Table 2) and was marginally lower in high-risk donors of the pre-DKT period (73.1 ± 2.0 years) as compared to that in all high-risk donors of the DKT protocol (i.e., SKT_hr_ and DKT donors, 75.5 ± 4.0, *P* < 0.01) or in DKT donors (Table 2).

### 3.2. Survival Analysis by Transplant Type

**Figure 4:**
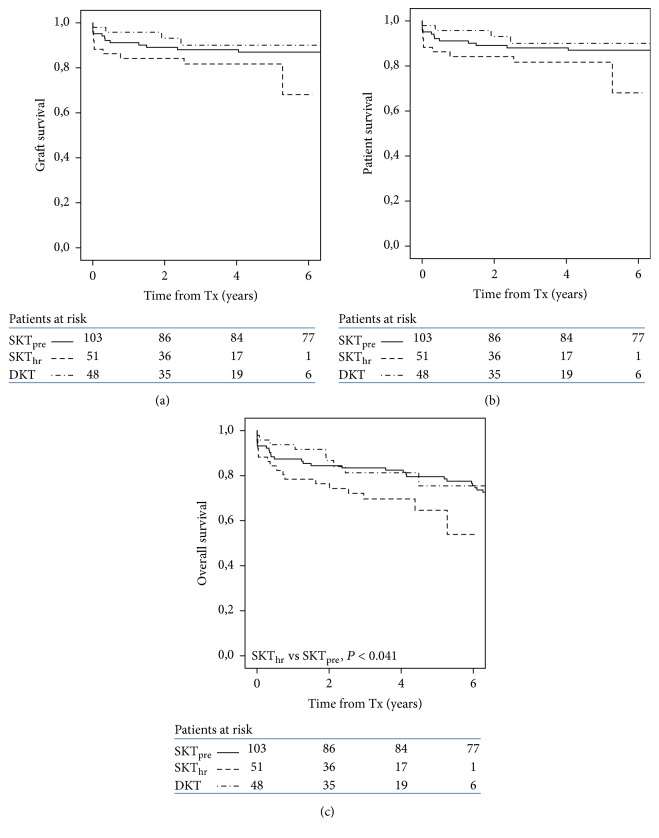
Kaplan-Meier plots of graft (a), patient (b), and overall survival (c) in recipients of high-risk ECD by transplant category. Overall survival is statistically lower in SKT_hr_ as compared to SKT_pre_.

Within the DKT protocol, there were no significant differences in graft, patient, and overall survival between recipients from ECD_lr_ and ECD_hr_ as a group (*P* < 0.516, 0.220 and 0.184, resp.), and between these 2 categories and SKT_pre_ (data not shown). Comparing individual transplant types from high-risk donors, there were no significant differences in graft and patient survival between SKT_hr_, DKT, and SKT_pre_, while overall survival was marginally lower in SKT_hr_ in comparison with SKT_pre_ (*P* < 0.041) (Figure 4). Among DKT recipients, 6 patients lost one graft (for venous thrombosis in 5 and arterial thrombosis in one), yet remaining graft maintained adequate kidney function along available follow-up in all 6. Score in these remaining grafts was 6 in 4 patients, and 5 and 3 in the other 2 patients (median score in remaining 40 patients with functioning DKT was 5, ranges 3–7).

**Figure 5:**
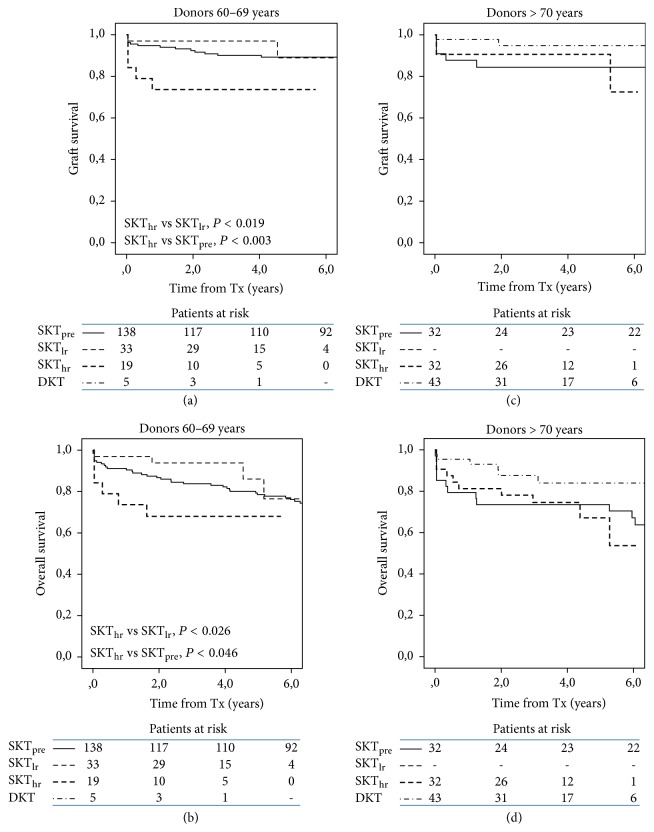
Kaplan-Meier plots of graft (a, c) and overall survival (b, d) in recipients by donor age and transplant category. Only in transplants from donors aged 60–69 years are graft and overall survival lower in SKT_hr_ as compared to SKT_lr_ and SKT_pre_.

Survival subanalysis by donor age showed that in the age range of 60–69 years SKT_hr_ had reduced graft and overall survival in comparison with SKT_lr_ (*P* < 0.019 and 0.026, resp.) and SKT_pre_ (*P* < 0.003 and 0.046, resp.) (Figures 4 and 5). DKT (only 5 cases in this donor age range) were not analyzed within this donor age category. In transplants from donors older than 70 years, there were no significant differences in graft and overall survival between SKT_hr_, SKT_pre_, and DKT.

### 3.3. Renal Function in Donors and Recipients

Renal function in donors and recipients of each transplant category is shown in Table 4. Donor plasma creatinine was similar in any transplant category, and eGFR was marginally lower in donors of DKT.

After transplantation DKT recipients showed better renal function and SKT_lr_ a lower one at 3 months, but at one year there were no statistical differences between all ECD transplant categories. Of note, in the 6 DKT patients with early loss of one graft creatinine clearance was 25 ml/min in 2 (with scores 3 and 5, resp.) and higher than 40 in all 4 patients with score 6 grafts.

### 3.4. Restricted Mean Number of Functioning Graft Years and Recipient Dialysis-Free Life Years

Table 5 shows that mean restricted number of functioning graft years by transplant reference at 1, 3, and 5 years after transplantation was equal in pre-DKT transplants and overall in the DKT protocol; in other words each patient transplanted from an ECD in the two periods had equal mean dialysis-free life years up to 5 years after transplantation. Calculation at the same times of total number of dialysis-free life years made possible by a given number of donors (e.g., 100) within each protocol showed a statistically significant higher quantity with the pre-DKT protocol at 3 and 5 years.

Since we did not know the discard rate of ECD organs in our regional agency before and after adopting the DKT protocol, we could only perform a quantitative analysis with reference to actual donors rather than to potential donors; however, taking into account the pre-DKT era acceptance and allocation criteria in our Centre, only one of all accepted donors in the DKT program (with a plasma creatinine 1.21 mg/dl and eGFR 48 ml/min/1.73 m^2^) would have not been accepted for SKT, indicating that rescue of marginal donor grafts through DKT option, in the context of a clinical and functional-based protocol, is not a frequent event.

## 4. Discussion

Our data shows that renal grafts from older donors, despite well-known inferior performance than grafts from younger donors, may offer satisfactory results: we observed a 5-year death-censored graft survival of 87.7% in the pre-DKT period and 84.2% in the DKT era, to compare with a 95.3% survival in recipients of donors younger than 60 years in our series (data not shown). This observation supports and encourages the acceptance of these donors for renal transplantation, even more so considering that median age of deceased donors in Italy and other western countries actually is older than 60 years [22, 23]. Our data also shows that adoption of a biopsy-based protocol for allocation of ECD grafts to SKT or DKT apparently did not result, in our case, in better overall results than our previous clinical protocol where organs were allocated to SKT according simply to morphological and functional suitability. “Overall” results include both early failures, which were not reduced in the biopsy protocol, and duration in time: indeed, at least in the short term (less than 6 years), death-censored and overall graft survival did not differ in the post-DKT period (biopsy-based) as compared to the pre-DKT period. Also the use of a novel method to compare effects of a given intervention in transplant outcome, that is, the mean restricted number of functioning graft years over a specified time interval [20, 21], showed equal results at 3 and 5 years for transplants in both the pre-DKT and the DKT protocols. This analysis allows a quantitative evaluation over time of transplant benefits in terms of overall dialysis-free patient life years for any quantity of actual donors. We have calculated that allocation criteria in use within the DKT protocol, with the observed blend of available donors, achieved a significantly lower quantity of total dialysis-free life years at 3 and 5 years than did an equal number of donors in the pre-DKT period with allocation to SKT according to clinical indication; in this calculation we have allowed for a conservative correction for unused grafts, which in our allocation area is about 1 in every 4 retrieved organs [22].

A possible bias in our analysis may be unequal “quality” of donors between the two periods, as suggested mainly by the age difference in donors of the two periods (pre-DKT and the DKT era), up to 8 years between mean age of high-risk donors in SKT_pre_ and in DKT, respectively. We tried to overcome this bias by performing a double subanalysis: first, we categorized pre-DKT donors according to the DKT protocol criteria in low-risk and high-risk donors and compared outcome data in the two periods within the same donor risk category. Second, we repeated survival analysis within overlapping donor age ranges for the two periods, that is, 60 to 70 years and older than 70 years. In both these subanalyses, we did not detect any significant differences in graft and overall survival between transplants from ECD of the two periods, in risk category and donor age ranges. Comorbidities were quite homogeneous between high-risk donors of both periods, and mean age was only 2.4 years lower in pre-DKT high-risk donors as compared to high-risk donors in DKT era. We think that this small difference has little impact on interpretation of data, even though we recognize that donors in the two periods were not fully homogeneous.

Surprisingly, biopsy and preimplantation histologic score in the DKT era did not prevent or reduce PNF incidence. Several conditions underlay PNF, intrinsic to both the organ (mainly its “senescence,” preservation mode, and time) and the recipient (vascular disease, rejection, early disease recurrence, etc.); in our cases we showed that organ quality was a main cause. Preimplantation score did not apparently differ in failed transplants and in those who performed well; since “chronic” lesions were much worse in posttransplant biopsy than in the preimplantation one, recipient factors, or inability of some organs to mount an adequate repair response to ischemia-reperfusion injury [24], might be considered, even though cold ischemia time and preservation mode did not differ in PNF cases as compared to the whole series. Pitfalls of biopsy itself may be considered: pathologists are well aware that detection and quantization of lesions of interest may greatly vary according to site, dimensions, mode of performing biopsy (core or wedge), sample processing, and even expertise of the pathologist itself [11, 25, 26]. Thus, the whole organ status may not be adequately represented by what is seen in actual biopsy. An indirect support to this contention was the equal distribution of PNF among scores 4, 3, or even 1-2. Others have already described poor correlation between preimplantation biopsy score and outcome of ECD grafts allocated to SKT [12, 25, 27].

Looking into the different transplant categories within the DKT protocol, there were no significant differences in graft survival between DKT and any SKT category (i.e., SKT_lr_ and SKT_hr_); restricting the analysis to high-risk donors, death-censored and overall graft survival were also equal in DKT and SKT_pre_ and marginally worst in SKT_hr_. Worst outcome in SKT_hr_ was mostly related to an unusually high rate of PNF in this transplant category, especially in the 60–69 donor age-range. In transplants from donors aged more than 70 years, there were no differences in graft, patient, and overall survival between DKT, SKT_hr_, and SKT_pre_. On the whole, our data shows that, at least in the short term, DKT do not appear to perform better than SKT from donors with equal comorbidity score and age range. Others have also shown equal survival of SKT and DKT from donors in the same age ranges (i.e., 60–69 and 70–79) [28]. Equal outcome with worst graft histology is commonly assumed to support the validity of DKT allocation by score [8, 14, 28]; contrary to this assumption are reports of good outcome of grafts with bad preimplantation biopsy, which would have indicated DKT according to the NITp protocol and are allocated to SKT without knowledge of biopsy data ([12, 13, 29] and personal data). We show here that allocation to SKT without biopsy of grafts from high-risk, older than 70 years, donors achieves similar survival than DKT from equally comorbid donors, provided that clinical suitability is carefully sought. We recognize that an apparent advantage of DKT over SKT in our series was a lower incidence of early failures; whether survival in time of DKT is also better than that of SKT from the same donor category and organs is actually uncertain. A relevant, non-biopsy-based study compared survival of DKT versus SKT from donors within equal quality score ranges, as defined by KDRI: it was shown that within any KDRI ranges overall survival was equal for grafts allocated to DKT or SKT except in the worst quality (higher KDRI) category, in which DKT showed a marginally better survival [30]; yet, also in this donor category transplants, the benefits, in terms of total dialysis-free patient life years, were less than half in DKT than in SKT.

As described by others [18, 31] we showed that DKT recipients who lost one graft maintained a satisfactory renal function in all the available follow-up (up to 76 months in a patient), despite a very bad histological score of remaining organ. This indicates that at least a fraction of grafts used for DKT might perform quite well as SKT in a double number of recipients.

“Best use” of organs is not easy to define, since in transplant activity scores and intended targets may have different perspectives: a “performance score” is primarily a recipient's, and possibly transplant Centre's (liable to performance rating) target: in this perspective allocation of high-risk donor organs to DKT confers better expectations of immediate and short term results. An “efficacy score” is instead a collectivity interest and should be intended to maximize years of dialysis-free life in recipients by available donors: in this perspective DKT reduces overall transplant number and possibly total dialysis-free patient life years. Discard rate should be included in efficacy score, which we did not dispose of; survival in a longer time is also a critical information, which requires a longer follow-up for meaningful comparisons and calculation of quantitative outcomes (i.e., survival data and dialysis-free life years). Equal survival of DKT and SKT, as suggested by KDRI categorization [30], needs to be confirmed in biopsy-based clinical studies. However, since patient death with functioning graft is a more frequent cause of graft loss than graft exhaustion in older recipients, the need for long-term graft survival might be less compelling in these recipients. As already discussed by others [29], we still need to define an “acceptable risk” of graft failure above which DKT may outperform SKT, or vice versa. On the basis of our data in the pre-DKT period, we favor the notion that, within a D-R age match, SKT may be considered as the default choice for most ECD, without need of biopsy if clinical data and organ anatomy appear permissive; DKT and biopsy should be limited to organs deemed unsuitable by clinical criteria, for example, with bad function and/or anatomy or very advanced donor age, in which case histology should be more directed to the acceptance/discard decision than to SKT/DKT allocation.

Our study has several limitations, the most relevant one being age difference between recipients and donors in pre-DKT and DKT era; however comparison within overlapping age ranges, with only 2.4-year difference in donor mean age, allowed homogeneous comparisons to be made. We also did not dispose of the discard rate in the two periods, a necessary information for an objective efficiency score appreciation. Lastly, number of transplants within the DKT protocol was small and follow-up in the DKT protocol was short, only allowing for “short-term” information and still awaiting for longer term confirmation.

In conclusion, our data show that use of a biopsy-based protocol to allocate grafts from aged donors to SKT or DKT may not result in better short and possibly long-term transplant outcome than a clinically based protocol with allocation only to SKT. In particular calculation of the mean number of functioning graft years by transplant showed equal year numbers at 1, 3, and 5 years in both the pre-DKT, clinical-based, and the DKT, biopsy-based, protocols but reduced total number of dialysis-free life years in recipients within the DKT protocol. DKT may confer some marginal advantage over SKT in reducing early graft failure rate but reduces the number of transplants and overall the total number of dialysis-free life years to patients, with still uncertain long-term transplant survival benefits.

## Figures and Tables

**Table 1:** Histologic score in use for kidney allocation to SKT or DKT of “high-risk” donors.

*Glomerular global sclerosis*	0: no glomeruli globally sclerosed
	1: less than 20%
	2: 20–50%
	3: >50%

*Arteries/arterioles wall thickness* ^*∗*^	0: normal appearance
	1: less than lumen diameter
	2: equal/slightly higher than lumen diameter
	3: higher than lumen diameter/severe lumen reduction

*Tubular atrophy*	0: absent
	1: less than 20% tubuli affected
	2: 20–50%
	3: >50%

*Interstitial fibrosis*	0: absent
	1: less than 20% parenchymal tissue substituted
	2: 20–50% tissue
	3: >50% tissue

^*∗*^The most severe lesion determines the score; the final score is the sum of 4 individual scores; with final score up to 4 (included) organs are allocated to solitary kidney transplantation (SKT); from 5 to 7 (included) organs are allocated to dual kidney transplantation (DKT); higher than 7 organs are discarded.

**Table 2:** Characteristics of donors older than 60 years according to period and transplant category.

Period	Pre-DKT^a^	DKT era^a^		
Tx category	SKT_pre_	SKT_lr_	SKT_hr_	DKT
*n*	170	33	51	48
M/F	84/86	18/15	26/25	27/21
Low-risk ECD, *n*	48	33		
(i) Age, yrs	64.2 ± 2.8	63.8 ± 2.5		
High-risk ECD, *n*	103		51	48
(i) Age, yrs^*∗*^	67.2 ± 4.7		71.2 ± 5.4	75.3 ± 4.8
(ii) Donors older than 70 yrs, *n* (%)	32 (31)		32 (63)°	43 (69)°
(iii) Donors older than 70 yrs, age	73.1 ± 2.0		74.4 ± 4.1	76.6 ± 3.7°
(iv) Arterial hypertension	55 (53)		23 (45)	30 (62)
(v) Diabetes	9 (9)		5 (10)	6 (12)
(vi) CV cause of death	60 (58)		17 (33)^+^	23 (48)

^a^“pre-DKT”, 1 Jan, 2000, to 30 Nov, 2010; “DKT era”, 1 Dec, 2010–31 Dec, 2015; SKT: solitary kidney transplant; suffix defines donor type: ECD: ECD from pre-DKT era, clinical risk undefined; lr, hr: ECD according to clinical low (no biopsy) or high risk (with kidney biopsy); DKT: dual kidney transplant; CV: cerebrovascular; ^*∗*^*P* < 0.0001 by ANOVA, and *P* < 0.05 or less by any intercategory comparisons; °*P* < 0.01 versus ECD_pre_; ^+^*P* < 0.05 versus ECD_pre_.

**Table 3:** Characteristics of recipients from donors older than 60 years according to period and transplant category and main transplant characteristics and outcomes. Data are numbers, mean ± SD, or median (ranges).

Period	Pre-DKT^a^	DKT era^a^		
Tx category	SKT_pre_	SKT_lr_	SKT_hr_	DKT
*n*	170	33	51	48
M/F	115/55	26/7	36/15	34/14
Age, years	59 ± 7.4	58.5 ± 8.7	63.5 ± 5.7^*∗*^	66.7 ± 4.5^*∗*^
Months on dialysis	45 (6–298)	50 (11–216)	37 (5–107)	29 (4–226)
1st-2nd-3rd Tx, *n*	152-15-3	29-4-0	50-0-1^+^	48-0-0^+^
HLA-MM^b^	4 (0–6)	4 (1–6)	4 (1–6)	5 (3–6)^*∗∗*^
Follow-up^c^, months/pt	72.0 (62.6–72.0)^*∗∗*^	44.1 (34.0–62.0)	43.3 (24.2–59.4)	44.2 (26.0–64.3)
Total follow-up, pt-yrs	827.4	126.7	169.6	176.0
CIT, hrs:min^d^	13:58 ± 4:08	14:04 ± 4:56	16:27 ± 3:53^*∗*^	16:05 ± 3:26^*∗*^
PNF, *n*. (surg)^e^	8 (4)	1	6 (2)	2 (2)
PNF, %	4.7	3.0	11.7	4.2
DGF, *n* (%)^f^	72 (42.3)	20 (60.6)	25 (49,0)	23 (47.9)
BPR-18mo, *n* (%)^g^	13 (7.6)	4 (12.1)	10 (20.8)^+^	4 (8.3)
Graft failure, *n* (*n*/100 pt-yr)^h^	21 (2.5)	3 (2.4)	10 (5.9)^+^	4 (2.3)
Death, *n* (*n*/100 pt-yr)	24 (2.9)	2 (1.6)	7 (4.1)	5 (2.8)

^a^Pre-DKT, DKT era: see Table 2. ^b^Mismatches on HLA loci A, B and DRB1; ^c^time to event or censoring; ^d^cold ischemia time; ^e^graft primary nonfunction (number of surgical failures in parenthesis); ^f^Delayed graft function (need for dialysis in the first week post-Tx); ^g^biopsy-proven rejection within 18 months after transplant; ^h^PNF and graft losses over time. Statistical differences: ^*∗*^*P* < 0.01 versus SKT_pre_ and SKT_lr_; ^+^*P* < 0.05 versus SKT_pre_; ^*∗∗*^*P* < 0.05 or less versus all other categories.

**Table 4:** Plasma creatinine (P_cr_, mg/dL) and GFR (Cockcroft-Gault formula in donors or 24 h-creatinine clearance in recipients) in donors (D) and recipients (R; at 3 and 12 months) in each transplant category (data are number, and mean ± SD).

	SKT_pre_	SKT_lr_	SKT_hr_	DKT	DKT_nfx_^ ^^a^
D-P_cr_	0.94 ± 0.19	0.92 ± 0.24	0.93 ± 0.17	0.90 ± 0.20	0.97 ± 0.26
D-eGFR	82.1 ± 20.9	85.9 ± 19.5	81.9 ± 24.9	73.8 ± 14.9^*∗*^	70.4 ± 11.8
*n*	170	33	51	48	6
R-P_cr_ 3 months	1.82 ± 0.79	2.32 ± 1.06^§^	2.01 ± 0.86	1.60 ± 0.70°	2.30 ± 0.43
R-C_Cr_ 3 months	46.6 ± 20.4	37.4 ± 16.7^§^	40.7 ± 17.2	49.6 ± 22.0^*∗∗*^	27.8 ± 11.2
*n*	156	32	44	45	6
R-P_cr_ 12 months	1.70 ± 0.65	1.77 ± 0.57	1.70 ± 0.51	1.60 ± 0.70	2.04 ± 0.62
R-C_Cr_ 12 months	48.0 ± 19.8	49.4 ± 21.6	48.8 ± 17.6	50.9 ± 20.3	37.2 ± 11.2
*n*	150	30	36	37	6

^a^6 DKT recipients with early removal of one graft; ^*∗*^*P* < 0.05 versus SKT_pre_ and SKT_lr_; ^§^*P* < 0.02 versus SKT_pre_; °*P* < 0.05 (or less) versus SKT_pre_, SKT_lr_, SKT_hr_, and DKT_nfx_^ ^; ^*∗∗*^*P* < 0.01 versus SKT_lr_, SKT_hr_, and DKT_nfx_.

**Table 5:** Mean restricted number (95% confidence intervals) of functioning graft years by transplant reference (MNFGY) at 1, 3, and 5 years after transplantation, and calculated total number of dialysis-free life years for every 100 donors (TNDFY) at the same times in recipients, in the pre-DKT and DKT protocols. Differences shown in bold indicate a significant statistical difference (*P* < 0.05).

	1 year	3 years	5 years
MNFGY			
Pre-DKT	0.93 (0.88–0.98)	2.62 (2.56–2.68)	4.33 (4.27–4.39)
DKT protocol	0.97 (0.92–1.02)	2.60 (2.53–2.67)	4.16 (4.08–4.25)
Difference	−0.04 (−0.14–0.06)	0.02 (−0.10–0.14)	0.16 (−0.02–0.31)

TNDFY			
Pre-DKT	149 (141–156)	419 (410–429)	693 (683–703)
DKT-protocol	134 (127–141)	359 (350–369)	576 (564–588)
Difference	15 (−1–30)	**60 (41**–**78)**	**117 (96**–**139)**

## Additional Points

Study protocols are registered at NITp-Centro Interregionale di Riferimento, Regione Lombardia, at n. P.T. 04-580, n. P.T. 02-580 (revised at n. P.T. 06-580).

## References

[1] Rosengard B. R., Feng S., Alfrey E. J. (2002). Report of the Crystal City meeting to maximize the use of organs recovered from the cadaver donor.

[2] Metzger R. A., Delmonico F. L., Feng S., Port F. K., Wynn J. J., Merion R. M. (2003). Expanded criteria donors for kidney transplantation.

[3] van Ittersum F. J., Hemke A. C., Dekker F. W. (2017). Increased risk of graft failure and mortality in Dutch recipients receiving an expanded criteria donor kidney transplant.

[4] Ojo A. O., Hanson J. A., Meier-Kriesche H. (2001). Survival in recipients of marginal cadaveric donor kidneys compared with other recipients and wait-listed transplant candidates.

[5] Merion R. M., Ashby V. B., Wolfe R. A. (2005). Deceased-donor characteristics and the survival benefit of kidney transplantation.

[6] Rose C., Schaeffner E., Frei U., Gill J., Gill J. S. (2015). A lifetime of allograft function with kidneys from older donors.

[7] Remuzzi G., Grinyo J., Ruggenenti P. (1999). Early experience with dual kidney transplantation in adults using expanded donor criteria. Double Kidney Transplant Group (DKG).

[8] Sefora P. E., Silvio S., Nicola D. F. (2013). Optimizing utilization of kidneys from deceased donors over 60 years: Five-year outcomes after implementation of a combined clinical and histological allocation algorithm.

[9] Fernández-Lorente L., Riera L., Bestard O. (2012). Long-term results of biopsy-guided selection and allocation of kidneys from older donors in older recipients.

[10] Stratta R. J., Farney A. C., Orlando G. (2016). Dual kidney transplants from adult marginal donors successfully expand the limited deceased donor organ pool.

[11] Liapis H., Gaut J. P., Klein C. (2017). Banff Histopathological Consensus Criteria for Preimplantation Kidney Biopsies.

[12] Foss A., Heldal K., Scott H. (2009). Kidneys from deceased donors more than 75 years perform acceptably after transplantation.

[13] Snanoudj R., Rabant M., Timsit M. O. (2009). Donor-estimated gfr as an appropriate criterion for allocation of ecd kidneys into single or dual kidney transplantation.

[14] Ruggenenti P., Silvestre C., Boschiero L. (2017). Long-term outcome of renal transplantation from octogenarian donors: A multicenter controlled study.

[15] Sung R. S., Christensen L. L., Leichtman A. B. (2008). Determinants of discard of expanded criteria donor kidneys: Impact of biopsy and machine perfusion.

[16] Nord Italia Transplant Program: Regione Lombardia Potocollo per l’utilizzo dei reni da donatoreanziano. http://www.policlinico.mi.it/AMM/nitp/area_operatore/protocolli/02/141230Rene_ProtocolloDonatoriAnziani.pdf.

[17] Nord Italia Transplant Program: Regione Lombardia Criteri per allocazione reni da donatore cadavere. http://www.policlinico.mi.it/AMM/nitp/area_operatore/linee_guida/02/RegoleGenerali/170224AllocazioneReniDaDonatoreCadavere.pdf.

[18] Cocco A., Shahrestani S., Cocco N. (2017). Dual kidney transplant techniques: A systematic review.

[19] Royston P., Parmar M. K. B. (2013). Restricted mean survival time: an alternative to the hazard ratio for the design and analysis of randomized trials with a time-to-event outcome.

[20] Pippias M., Jager KJ., Caskey F. (2017). Kidney transplant outcomes from older deceased donors: A paired kidney analysis by the ERA-EDTA Registry.

[21] Lim W. H., Chang S., Chadban S. (2010). Donor-recipient age matching improves years of graft function in deceased-donor kidney transplantation.

[22] Centro trasfusionale e di immunologia dei trapianti, Ospedale Maggiore di Milano, Policlinico IRCCS. http://cm.argonet.it/websites/policmi/staging/home_ctit.nsf/wAssets/IDCW-8HTBUL/$file/Report%20NITp%20maggio%202011.pdf.

[23] Matesanz R., Domínguez-Gil B., Coll E., Mahíllo B., Marazuela R. (2017). How Spain Reached 40 Deceased Organ Donors per Million Population.

[24] Woo Y. M., Gill J. S., Johnson N., Pereira B. J. G., Hariharan S. (2005). The advanced age deceased kidney donor: Current outcomes and future opportunities.

[25] Wang C. J., Wetmore J. B., Crary G. S., Kasiske B. L. (2015). The donor kidney biopsy and its implications in predicting graft outcomes: A systematic review.

[26] Azancot M. A., Moreso F., Salcedo M. (2014). The reproducibility and predictive value on outcome of renal biopsies from expanded criteria donors.

[27] Sánchez-Escuredo A., Sagasta A., Revuelta I. (2017). Histopathological evaluation of pretransplant donor biopsies in expanded criteria donors with high kidney donor profile index: a retrospective observational cohort study.

[28] Messina M., Diena D., Dellepiane S. (2017). Long-term outcomes and discard rate of kidneys by decade of extended criteria donor age.

[29] Hofer J., Regele H., Böhmig G. A. (2014). Pre-implant biopsy predicts outcome of single-kidney transplantation independent of clinical donor variables.

[30] Klair T., Gregg A., Phair J., Kayler L. K. (2013). Outcomes of adult dual kidney transplants by KDRI in the United States.

[31] Cruzado J. M., Fernandez L., Riera L. (2011). Revisiting double kidney transplantation: Two kidneys provide better graft survival than one.

